# Editorial: Harnessing machine learning to decode plant-microbiome dynamics for sustainable agriculture

**DOI:** 10.3389/frmbi.2025.1602938

**Published:** 2025-06-03

**Authors:** Mohsen Yoosefzadeh Najafabadi, Eman M. Khalaf, Mohamed Mysara, Ahmed M. El-Baz

**Affiliations:** ^1^ Department of Plant Agriculture, University of Guelph, Guelph, ON, Canada; ^2^ Department of Microbiology and Immunology, Faculty of Pharmacy, Damanhour University, Damanhour, Egypt; ^3^ Bioinformatics Group, Center for Informatics Science, School of Information Technology and Computer Science, Nile University, Giza, Egypt; ^4^ Department of Microbiology and Immunology, Faculty of Pharmacy, Delta University for Science and Technology, Gamasa, Egypt

**Keywords:** crop disease detection, crop yield improvement, machine learning algorithms, plant microbiome, phenotypic traits

The world’s growing population of nine billion people is facing a severe global food insecurity crisis, especially in low and middle-income countries (Hong et al., 2022). Improving crop yield and productivity through structured breeding programs is a key strategy to address this issue (Yoosefzadeh-Najafabadi et al., 2024). Plants and microbes have evolved intricate relationships over millennia, providing benefits such as enhanced growth, improved nutrient uptake, and increased stress tolerance to plants (Trivedi et al., 2022). In recent years, research has focused on the interplay between the plant microbiome and phenotype to enhance breeding programs (Nerva et al., 2022; Araujo et al., 2024; Batool et al., 2024).

Traditional analysis methods struggle to handle data from high-throughput technologies such as meta-genomics, meta-transcriptomics, and meta-proteomics (Yoosefzadeh Najafabadi and Torkamaneh, 2025), leading to a lack of understanding of how the microbiome influences plant traits (Trivedi et al., 2022). Advanced data analysis techniques have been developed to integrate and analyze data from multiple omics sources effectively (Trivedi et al., 2022). To harness the potential of plant microbiomes, researchers are increasingly turning to machine learning, a subset of artificial intelligence that enables computers to learn from data and make predictions (De Souza et al., 2020). Deep-learning models, a powerful type of machine learning, are particularly effective for analyzing complex biological data. These models are built from layers of interconnected nodes that process input data, such as microbial DNA sequences or plant images, to identify patterns and relationships. Developers must make critical decisions when designing these models, such as choosing the number and type of layers, selecting the data features to focus on (e.g., specific microbial traits), and determining how the model learns from errors (Zhou and Gallins, 2019). These choices depend on the specific problem, such as detecting crop diseases or predicting yield, and are guided by the need for accuracy, computational efficiency, and applicability to real-world farming conditions (Zhou and Gallins, 2019).

The development of a machine vision-based method using an enhanced YOLOv5s model for grading individual peanut pod rot, which is a major plant disease affecting peanut production were investigated in a recent paper published by (Liu et al.) YOLO is a real-time object detection algorithm known for its speed and efficiency. Unlike traditional methods that repurpose classifiers or localizers to perform detection, YOLO frames object detection as a single regression problem, directly predicting bounding boxes and class probabilities from full images in one evaluation. This model, which relies on deep-learning principles to process images, incorporates a Shuffle Attention module to focus on key visual features and replaces the loss function CIoU with EIoU to improve accuracy in distinguishing non-rotted and rotten peanuts in complex backgrounds. The study also highlighted the potential for future research to enhance prediction performance for different peanut varieties and to consider factors like rotten kernel rate for better yield estimation. In another study by Pandiyaraju et al., the possibility of using a machine vision-based approach for grading individual peanut pod rot using an improved YOLOv5s algorithm were investigated. The study addresses the challenges of visually identifying and classifying peanut pod rot by introducing a Shuffle Attention module to enhance feature representation and accuracy in complex backgrounds. The proposed model demonstrated high recognition rates for non-rotted and rotten peanuts, offering a promising solution for automated grading of peanut pod rot, providing advancements in disease resistance evaluation and germplasm selection in peanut breeding. Another use of YOLO algorithms was reported by Wang et al. where they enhanced the identification of potato seedlings in drone-acquired images by introducing a new lightweight model named VBGS-YOLOv8n. By utilizing a modified version of YOLOv8n with a lighter backbone network and incorporating improvements such as a bidirectional feature pyramid network and GSConv and Slim-neck designs, the model achieves high precision and detection performance.

Precise identification and enumeration of flax plant organs play a vital role in acquiring key phenotypic data necessary for selecting and managing flax varieties. In research conducted by Kai et al., a Flax-YOLOv5 model is presented to extract phenotypic information from flax plants. By extending the YOLOv5x network with the BiFormer module, which integrates bi-directional encoders and converters to focus on essential features adaptively, the model’s computational efficiency is enhanced. Zhang et al. introduced a novel method for detecting small target cotton bolls in cotton fields using unmanned aerial vehicle (UAV) imagery. By employing the YOLO SSPD model, which integrates space-to-depth convolution and a small target detector head, the researchers achieved significant improvements in boll detection accuracy on UAV imagery. The model demonstrated high precision and efficiency in detecting cotton bolls, supporting the cotton production process and enhancing reliability in yield estimates. In another research conducted by Tang et al. they tried to overcome the issues related to low detection accuracy and limited applicability across different ripeness levels and varieties of large non-green-ripe citrus fruits in complex environments. The study introduces YOLOC-tiny, a precise and lightweight model based on YOLOv7 that leverages EfficientNet-B0 as the feature extraction backbone. To enhance detection performance, a convolutional block attention module (CBAM) is integrated into the aggregation network, along with an adaptive intersection over union regression loss function tailored to large non-green-ripe citrus characteristics. Furthermore, a layer-based adaptive magnitude pruning technique is utilized to reduce redundancy in model parameters. In practical applications such as fruit-picking robots, YOLOC-tiny achieves a high accuracy of 92.8% at a swift frame rate of 59 frames per second. (Wang et al.) also introduced an improved target detection and pose estimation model called PAE-YOLO for identifying Xiaomila fruits in complex farmland environments. The model combines an EMA attention mechanism and a DCNv3 deformable convolution module to enhance feature extraction capability and reduce computational complexity. Experimental results show that the PAE-YOLO model outperforms other classic detection models in terms of accuracy, model size, and computational efficiency. The model achieved an average mean accuracy of 88.8% and a F1 score of 83.2%, with improved performance in target detection and posture estimation.

**Figure 1:**
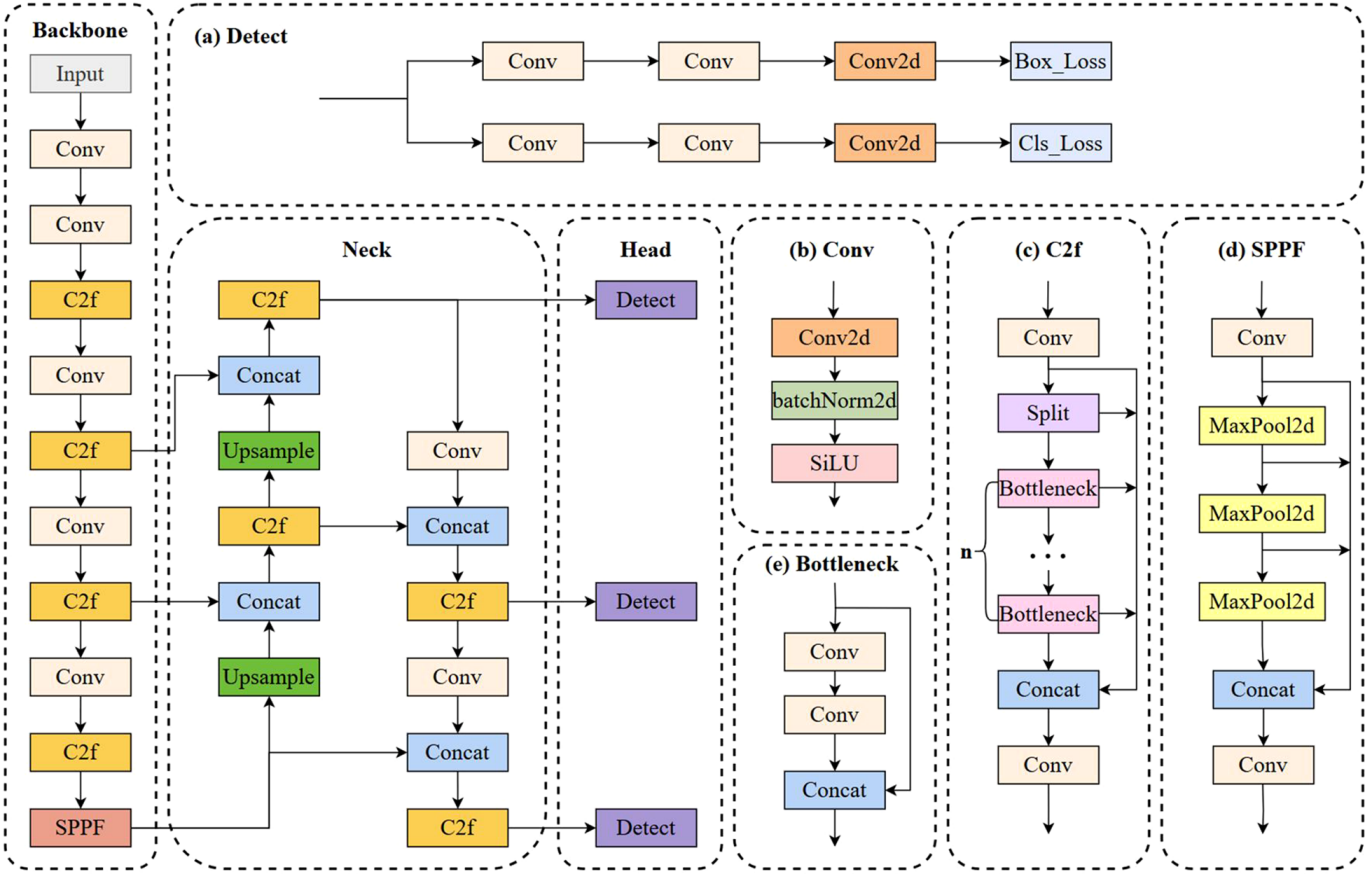
YOLOv8 algorithm model. The network consists of three main components: the Backbone, Neck, and Head. It incorporates several functional modules, including the detection head **(a)**, convolutional block **(b)**, cross stage partial with bottlenecks (C2f) **(c)**, spatial pyramid pooling–fast (SPPF) **(d)**, and bottleneck module **(e)**. Source: Reproduced from Sun.

Efficiently detecting tomatoes in complex environments is important for automating tomato harvesting. The proposed S-YOLO model by Sun, an enhancement of YOLOv8s, introduces innovations such as a lightweight GSConv_SlimNeck structure, improved α-SimSPPF and β-SIoU algorithms, and an SE attention module to boost detection accuracy and speed (**Figure 1**). Experimental results show the S-YOLO model achieves 96.60% accuracy and 74.05 FPS, outperforming previous models and making it ideal for use in robotic tomato-picking systems. In a study conducted by Liu et al., the YOLO-SwinTF proposed based on YOLOv7, incorporates Swin Transformer blocks for capturing global visual information and Trident Pyramid Networks for improved feature communication. The model uses Focaler-IoU to adjust focus on sample distribution. Tested on a tomato dataset, it achieved higher recall, precision, F1 score, and AP compared to YOLOv7, showing strong robustness in challenging conditions and improved detection accuracy without compromising speed.

Plant diseases pose a significant threat to global agriculture by negatively impacting crop yield and quality (Yoosefzadeh Najafabadi, 2021). Despite the challenges associated with identifying and classifying these diseases, a new approach leveraging deep learning algorithms and convolutional neural networks (CNNs) has been proposed to accurately detect and categorize leaf diseases in economically important crops such as strawberries, peaches, cherries, and soybeans (Prince et al.). For this aim, a research focuses on categorizing 10 disease classes for these crops, comprising 6 diseased classes and 4 healthy classes, using a CNN-support vector machine (SVM) model (Prince et al.). Various pre-trained models were employed, with the proposed model achieving an average accuracy of 99.09%, outperforming established models like VGG16. The model utilizes Class Activation Maps generated through the Grad-CAM technique to visually illustrate detected diseases and produce heatmaps highlighting the areas requiring classification (Prince et al.). The FCHF-DETR model developed by Xin and Li, an enhancement of RT-DETR-R18, addressed the challenges of detecting tomato leaf diseases with FasterNet, Cascaded Group Attention, and HSFPN. Using a dataset of 3147 images, the model achieved high precision and recall while reducing computational demands. In addressing the challenge of identifying tea plant diseases amidst complex backgrounds, the ECA-ResNet50 model improved the ResNet50 architecture by using a multi-layer small convolution kernel strategy and introducing the ECA attention mechanism (Li and Zhao). This enhances feature extraction, achieving a 93.06% accuracy rate, a 3.18% improvement over the original model. The model’s strong generalization capabilities indicate its effectiveness in mitigating background interference and precisely recognizing tea disease targets across various plant datasets (Li and Zhao).

Chinese Herbal Medicine (CHM) faces automation challenges in microscopic identification due to traditional method limitations and dataset issues. In a study developed by Zhu et al. introduced a deep learning-based approach, employing segmentation-combination data augmentation and a shallow-deep dual attention module to enhance feature focus. The CHMMI approach achieves high precision and outperforms models such as YOLOv5 and ResNet, offering a robust solution to modernize CHM identification. Jia et al. proposed an enhanced DeepLabv3+ model, named DFMA, incorporating a novel PSPA-ASPP structure for efficient phenotyping analysis. Tested on various datasets, the model achieved high mIoU scores, outperforming existing models. It provides detailed segmentation and precise seedling measurements, offering an automated solution to improve analysis efficiency and overcome traditional method challenges.

Potatoes are known as one of the staple foods globally, and timely detection of foliar diseases is essential for healthy yields. Traditional image classification struggles with inconsistent data, so a new model combines EfficientNet-LITE for feature extraction with KE-SVM Optimization for classification. The method developed by Sangar and Rajasekar refined accuracy by cross-referencing misclassifications, achieving improved accuracy (87.82% for uncontrolled data and 99.54% for controlled data) while maintaining computational efficiency. The model’s small size and low floating point operations per second (FLOPs) make it ideal for mobile and edge devices, enhancing its practical use in precision agriculture. Hyperspectral images provide detailed information, important for classifying corn seed varieties with different internal structures. Existing methods struggle with feature extraction from these complex datasets, resulting in low accuracy (Wang et al.). To overcome this, the spectral-spatial attention transformer network (SSATNet) is proposed by Wang et al., which utilizes 3D and 2D convolutions for feature extraction and incorporates a transformer encoder with cross-attention for global perspective refinement. This approach improves classification performance on hyperspectral corn image datasets, demonstrating its effectiveness over current methods.

Despite the transformative potential of machine learning in analyzing plant-associated microbiomes, several challenges persist. High-quality, standardized datasets are often scarce, particularly for underrepresented crops or regions, limiting model generalizability. Scalability remains a hurdle, as many models require significant computational resources, which may not be accessible to small-scale farmers or researchers in low-resource settings. Additionally, integrating multi-omics data with environmental and phenotypic variables across diverse agricultural systems is complex, often leading to inconsistent predictions. These limitations highlight the need for robust, adaptable frameworks that can accommodate varied data types and practical constraints. Looking forward, promising directions include fostering interdisciplinary collaborations between plant scientists, data scientists, and farmers to develop user-friendly tools that bridge research and application. Advances in computational efficiency, such as lightweight models and edge computing, could democratize access to machine-learning technologies. Furthermore, field-based validations and longitudinal studies are essential to ensure models perform reliably under real-world conditions. By addressing these challenges and leveraging emerging technologies, the scientific community can unlock the full potential of plant microbiomes to enhance crop resilience and global food security.
